# A five-year longitudinal observational study in morbidity and mortality of negative appendectomy in Sulaimani teaching Hospital/Kurdistan Region/Iraq

**DOI:** 10.1038/s41598-020-58847-1

**Published:** 2020-02-06

**Authors:** Hiwa Omer Ahmed, Rizgar Muhedin, Amir Boujan, Aso Hama Saeed Aziz, Ara muhamad Abdulla, Rezan Ahmed Hardi, Aso Ahmed Abdulla, Taban Aziz Sidiq

**Affiliations:** 1 College of Medicine, University of Sulaimani, Sulaimani city, Kurdistan region, Iraq; 2 Surgery Department, Sulaimani Teaching Hospital, Sulaimani city, Kurdistan region, Iraq; 3 Emergency Department, Sulaimani Teaching Hospital, Sulaimani city, Kurdistan region, Iraq; 4 Gynecology and obestetrics, Gynecology and obestetrics hospital Sulaymaniyah, Sulaimani city, Kurdistan region, Iraq

**Keywords:** Intestinal diseases, Epidemiology

## Abstract

The most common surgical emergency is suspected acute appendicitis, the lifetime risk of acute appendicitis is estimated to be 8.6% for men and 6.7% for women, with a male to female ratio of 1.4:1; correct diagnosis can be made in 70–80% of patients after the operations about 32% of appendectomies revealed normal appendices and meanwhile appendectomy has a considerable morbidity and mortality. The aim is to explore potential morbidity and mortality associated with negative appendectomy. Prospective case series study, including 5847 patients, who were suspected to have acute appendicitis over a period of five years from 1st December 2013 to 30th November 2018, in emergency department of Sulaimani Teaching Hospital. All the collected data were collected, organized then analyzed by Statistical Package for the Social Sciences version 21. Morbidity in the patients with negative appendectomies occurred in patients in the form of 90 (01.91%) wound infection, 48 (01.02%) intestinal obstruction and last 15 (00.32%) patients developed septicemia. While mortality in negative appendectomy patients was 21, (00.45%). Negative appendectomies have high rates of morbidity and mortality, knowing real rates may help in considering various policies and may be helpful to elude avoidable complications and potential mortality.

## Introduction

Non traumatic acute abdomen is 4–8% of adults admitted to the emergency department^1,2^. The most common surgical emergency is suspected acute appendicitis, “the lifetime risk of acute appendicitis is estimated to be 8.6% for men and 6.7% for women”^3^. Males are affected one and half more times than females^4^ while definite diagnosis could be done in 70–80% of patients^1^, after the operations about 32% of appendectomies revealed normal appendices^5^,

In females of child bearing age, gynecological pathologies are simulating acute appendicitis^1^, which may increase the prevalence of negative appendectomies.

Clinical suspicion of acute appendicitis is mostly done on patient’s symptoms and physical findings, including Alvarado score “which has adding accuracy (98.60%) versus computerized tomography (CT) scan (99.03%)^6^. Although laboratory blood and urine investigations and simple imaging like plain erect abdominal radiogram and ultrasonography of abdomen and pelvis may aid in accuracy of the diagnosis. “But are not as helpful as magnetic resonance imaging (MRI) and CT scan, which have accuracy of 98% in diagnosing acute appendicitis and lastly diagnostic laparoscopy”^1,3,7–10^.

These advanced modalities of diagnosis may not be available out the hours of daily work especially at night and in primary surgical centers, particularly in the developing countries, “Whenever they are present, usually reserved for moderate-risk patients whose diagnoses are often ambiguous”^3^. Nonetheless, in countries with insufficient healthcare resources, the widespread use of CT may not be cost-effective. Therefore, it is also necessary for the emergency department to spend time for such investigations^10^.

The Alvarado score is “frequently been used, and it has been validated to be simple, practical diagnostic tools for accessing acute abdomens”^1,3,11^, which is deduced from history, physical finding and simple laboratory investigations. Ultrasound is safe and available, it may show ultrasonic features of acute appendicitis; “which are diameter > 6 mm, lack of compressibility, hyperemia of the appendicular wall, peri-appendiceal inflammatory changes, and the presence of peritoneal fluid with the additional benefit of suggesting an alternative diagnosis for acute abdominal pain in up to two-thirds of patients”^12^.

While in patients with classic signs and symptoms, the clinical diagnosis may be clear, atypical manifestations that lead to diagnostic confusion and delay in treatment^12,13^.

The treatment of acute appendicitis remains a health problem, given all medical advances^13^, and significant morbidity (10%) and mortality (1–5%) are still associated with it^14^.

The common standard of care for appendicitis patients is the laparoscopic or open surgical appendectomy. In some cases, a non-operational antibiotic strategy is beneficial and emerging research indicates that broader applicability may occur^15^.

About one fifth to one third of appendectomies are negative and revealing normal appendices^5,16,17^, adverse outcomes and mortality after the operations of negative appendectomies are loosely defined in the literature, the work is aiming to explore potential morbidity and mortality associated with negative appendectomy.

## Results

### Age groups & gender difference

The most common age groups for acute appendicitis were (21–30 and 31–40 years), with gender ratio F/M = 1.49/1.00 as shown in Table 1.

**Table 1:** Age groups of the patients admitted as suspected acute appendicitis.

Age groups	Frequency & percentage		Total (n = 5847)
	♀	♂	
12–20	539 15, 36%	359 15.34%	898 15.35%
21–30	1340 38.19%	893 38.17	2233 38.19%
31–40	1346 38.36%	897 38.35	2243 38.36%
41–50	283 08.06%	114 04.87%	397 06.78%
	**Total** **(n = 3508)**	**Total** **(n = 2339)**	**Total** **(n = 5847)**

The most common age group for suspected acute appendicitis was 31–40 years of age, with female predominance female/male (F/M) ratio in this age group was 1.5/1.0.

From a total of 1073 (22.82%) patients having negative appendectomy, half (648, 14.55%) of these patients had no any other pathologies. Meanwhile the rest had one of the surgical or gynecological and obstetric pathologies; i.e.75 (1.59%) patients had other abdominal alternative diagnosis, composing of (n = 49, 01.04) patients with mesenteric lymphadenitis, urinary tract infection (n = 7, 00.14), ureterolithiasis (n = 4, 00.09%), epiploic appendage torsion (n = 6, 00.13%), gastroenteritis (n = 4, 00.09%) and perforated duodenal ulcer (n = 2,), omental torsion (n = 2, 00.04%), Crohn disease (n = 1, 00.02%),

### Acute and negative appendectomies

Acute appendicitis were found in (3628, 77.18%) patients from a total of 4701 patients with suspension of acute appendicitis, normal Michel diverticulum were found in 11(00.23%) patients with acute appendicitis, while Michel Diverticulitis were found in 3 patients (00.06%) with normal appendices, the percentage of normal appendices was (n = 1073, 22.82%), see Table 2.

**Table 2:** Final diagnosis and histopathological results of the patients admitted as suspected acute appendicitis in each age groups.

Variables			♀ (n = 3155)	♂ (n = 1546)	Total and % (n = 4701)
**Acute appendicitis** (n = 3628, 77.18%)			2835 60.31%	793 16, 87%	3628 77.18%
**Normal appendices** **(n = 1073, 22.82%)**	**with Surgical pathologies**	*Other abdominal alternative Diagnosis	32 00.68%	18 00.38%	50 01.80%
		**Michel Diverticulitis**	1 00.02%	2 00.4%	3 00.06%
	**without other pathologies**		428 09.10%	220 04.67%	648 26.08%
	**Gynecological and obstetric pathologies**	**Ruptured Graafian follicle (Mittelschmerz)**	125 02.65%	0 00.00%	125 02.65%
		**Ovarian cyst**	95 02.02%	0 00.00%	95 02.02%
		**Hemorrhage into an ovarian cyst**	74 01.57%	0 00.00%	74 01.57%
		**Acute pelvic inflammatory disease**	53 01.13%	0 00.00%	53 01.13%
		**Endometriosis**	18 00.38%	0 00.00%	18 00.38%
		**Ectopic Pregnancy**	7 00.15%	0 00.00%	7 00.15%

### Gynecological alternative diagnosis

The most common gynecological pathologies were ruptured Graafian follicle in (n = 125, 02.65%) patients, followed by ovarian cyst, hemorrhage into an ovarian cyst, acute pelvic inflammatory disease, endometriosis, and ectopic pregnancy (02.02%), (01.57%%), (01.13%), (00.38%), and (00.15%) respectively.

### Morbidity

Morbidity occurred in 875 (18.61%) patients, who underwent appendectomy as follows: 601, (12.78%) of them developed wound infection and 216, (04.59%) patients developed intestinal obstruction, most responded well to the conservative management, while 58 (01.23%) patients developed septicemia. Fifty eight, (01.23%) of the patients deteriorated and died within first month after the operation, shown in Table 3.

**Table 3:** Morbidity in patients having acute or negative appendectomies.

Morbidity and mortality	Acute appendicitis (n = 3128)		Negative appendectomy (n = 1573)		Total (n = 4701)	P-value
	♀	♂	♀	♂		
Wound infection	311 06.61%	200 04.25%	49 01.04%	41 00.88%	601 12.78%	0.028424
Intestinal obstruction	91 01.93%	77 01.63%	19 00.40%	29 00.62%	216 03.69%	
Septicemia	23 00.48%	20 00.43%	7 00.15%	8 00.17%	58 01.23%	
Total	425 09.05%	297 06.32%	75 01.59	78 01.65%	**875** 18.61%	

Morbidity in the patients with negative appendectomies occurred in patients in the form of 90 (01.91%) wound infection, 48 (01.02%) intestinal obstruction and last 15 (00.32%) patients developed septicemia. While mortality in negative appendectomy patients was 21, (00.45%) see Table 4. Thirteen (00.28%) patients died from sepsis, four (00.08%) patients developed severe pulmonary embolism, then passed away, two (00.04%) were died after multi-organ failure because of small intestinal obstruction, and two (00.04%) patient not recovered from anesthesia, see Table 4.

**Table 4:** Mortality in patients having acute or negative appendectomies.

Mortality	Acute appendicitis (n = 3128)		Negative appendectomy n = 1573)		Total (n = 4701)	P-value
	♀	♂	♀	♂		
Total	23 00.49%	14 00.29%	12 00. 26%	9 00.19%	58 01.23%	0.026149.
sepsis	11 00.23%	11 00.23%	7 00.15%	6 00.13%		
Multi-organ failure	7 00.15%	1 00.02%	1 00.02	1 00.02%		
Pulmonary embolism	3 00.06%	1 00.02%	3 00.06%	1 00.02%		
Not recovered from anesthesia	2 00.04%	1 00.02%	1 00.02%	1 00.02%		

Commonest morbidity in both genders, both in acute appendicitis and negative appendectomy was wound infection in the form of surgical site infection (n = 601, 12.78%); ninety (01.91%) patients had negative appendectomy, and 511 (10.87%) had acute appendicitis, Table 3.

While intestinal obstruction occurred in 216 (04.59%) of the total of 4701 patients with suspected acute appendicitis; forty eight (01.02%) had negative appendectomy, and 168 (03.57%) patients had acute appendicitis.

**Figure 1:**
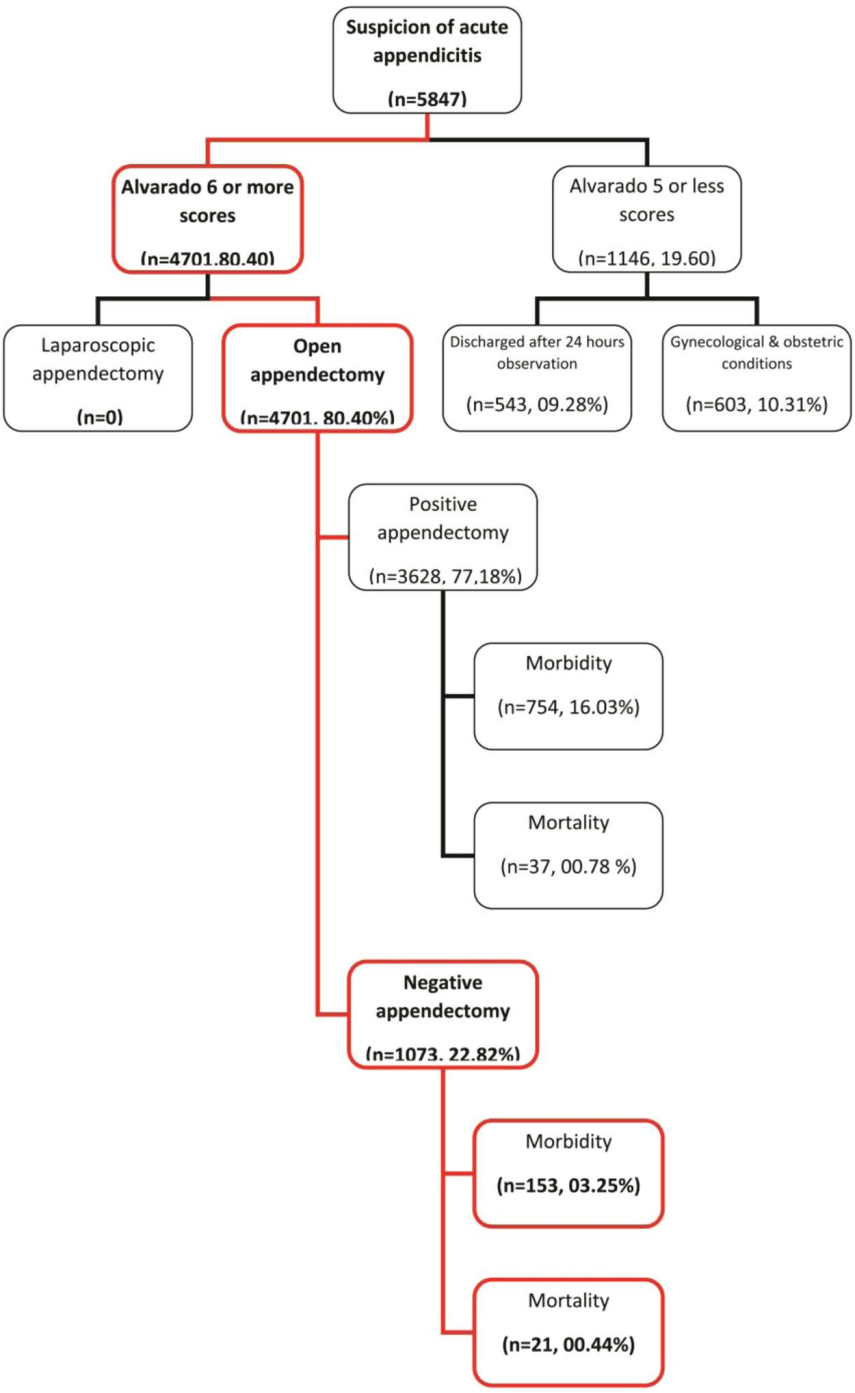
The total number of the patients who were admitted on suspension of acute appendicitis and their flow in the emergency department.

The least common morbidity was septicemia, found in 58 (01.23%) of all the patients underwent appendectomy, fifteen (00.32%) has normal appendices, and forty three (00.91%) patients had acute appendicitis. The chi-square statistic, p-value = 0.028424, it is significant statistically at p < 0.05. This low P value suggests that the results provides enough evidence that; the null hypothesis (against hypothesis which declaring significant of comorbidity in negative appendectomy) might be rejected, revealing significant association between negative appendectomies and significant rates of potential post-operative morbidities, look to Table 3. Figure 1. This is clarifying that patients with negative appendectomy had higher morbidity as compared to patients with acute appendicitis. Figure 1 showing the total number of the patients who were admitted on suspension of acute appendicitis and their flow in the emergency department.

### Mortality

There were fifty eight (01.23%) deaths in the first month after appendectomies, twenty three (00.49%) patients underwent appendectomies, found to have normal appendices died versus thirty seven (00.78%) patients, had acute appendicitis. The commonest cause of death was sepsis, followed by multi-organ failure, pulmonary embolism, unrecovered from general anesthesia. The chi-square statistic, p-value = 0.026149 and significant statistically at p < 0.05. This small p-value indicates strong evidence against the null hypothesis, which may means that mortalities occurred after negative appendectomies are significant statistically Table 4, Fig. 1. This is clarifying that patients with negative appendectomy had higher mortality as compared to patients with acute appendicitis

## Discussion

Acute appendicitis is a serious surgical emergency, especially during the early stages of the disease, diagnosis is not straightforward^3^. Many diagnostic pitfalls remain, which can be associated with a substantial number of misdiagnoses and/or avoidable surgery^2,18^. The price of postponement or^3^ Inappropriate diagnosis may be huge, with significantly higher morbidity and mortality rates in addition to severe appendicitis^15^.

While appendectomy has been considered the gold standard, more and more recognition is gaining in recent conservative antibiotic management^19,20^. Many patients may experience an episode that will not progress or may even be self-limiting, and sometimes antibiotics alone will be necessary, successful and safe^20^. and Valid non-surgical solution for uncomplicated appendicitis. The patient who had low clinical suspicion of acute appendicitis (Alvarado ≤ 5) and were monitored with IV antibiotics, no one of them required surgery due to lack of improvement on conservative management.

The commonest age group for suspected acute appendicitis was (31–40), with female predominance, F/M ratio was 1.49/1 Tables (1 and 3), as there are gynecological pathologies mimicking acute infection of vermiform appendix.

From a sum of 4701 ill persons, who underwent appendectomy, there were (n = 1073, 22.82%) negative appendectomies, look to Table 2. Osime O., *et al*.^21^ found the incidence of negative appendectomy to be about 16.1%,

Ratio of F/M in negative appendectomies was 3.43/1 as shown in Table 2, this is in line of literature as Osime O., *et al*.^21^ stated that “female had more negative appendectomy”

In the current work morbidity and mortality were higher in acute appendicitis; morbidity was (n = 722, 15.37%), mortality was (n = 37, 00.78%) while in negative appendectomies; morbidity was (n = 153, 02.87%), mortality (n = 21, 00.45%) as shown in Tables 3 and 4.

While Lee, M., *et al*.^22,23^ “found complication rates of appendectomy for inflamed appendix (16.7%), and for a negative appendectomies (14.2%).

Female have higher rate of negative appendectomy, and associated with higher complications after negative appendectomy versus males; this is going with others results^17,22,24^.

Female also have higher mortality after negative appendectomy in comparison to male patients (00.26% versus 00.19%), this is in contra to others study, Seetahal SA., *et al*. states^17^ that” men associated with higher rate of mortality reaching (1.93%), but Lee, M. *et al*., and Ferris, Mollie, *et al*.^22,23^ found “lower complication rate in male patients (5.4%).

To decrease negative appendectomy, and postoperative complication “accurate and timely diagnosis are needed”^15,25^ and perforated appendicitis in most of the patients^26^, is a result of delayed consultation, additional ultrasonography or CT scan of the abdomen can be performed in order not to operate hastily, albeit it is recommended to monitor changes in symptoms a little longer^27^, with retaking precise symptoms and signs catlike observation of 12 ± 8 hours could be sufficient to make surgical decisions, “Despite a high negative appendectomy”^17,28^.

The frequency of complications in the negative and positive groups and the rate of death from the removal of a normal appendix are also broadly similar^22^.

As newly industrialized region with increasing incidence of appendicitis we need to be more meticulous in diagnose and management of suspected acute appendicitis or “otherwise risk unnecessary morbidity and mortality”^23^ after negative appendectomy.

### Patients, materials and methods

Prospective case series study, including 5847 patients, who were suspected to have acute appendicitis over a period of five years from 1^st^ December 2013 to 30^th^ November 2018, in emergency department of Sulaimani Teaching Hospital. This is a tertiary surgical hospital in Sulaimani city, Kurdistan, Iraq, draining about 2 million peoples in the Governorate of Sulaimani. All methods were performed in accordance with the relevant guidelines and regulations, and the work was approved by Ethics Committee of college of Medicine, University of Sulaimani (No. 2, on 8^th^, November 2013). Informed consent were explained and signed by the patient and senior house officer on duty. While for human participants under the age of 18 years informed consent was obtained from a parent and/or legal guardian for study participation^29–31^.

All demographic, clinical including Alvarado score, laboratory, imaging, operative data, were collected incognito by filling a questionnaire.

The diagnosis were mostly clinical,, The patients were assessed with thorough medical history, physical examination with some simple investigations (general urine examination, complete blood count, plain erect abdominal radiogram, and when available, the patients were sent for ultrasonography of the abdomen and pelvis, while some patients were sent selectively for beta human chorionic gonadotropin (ß-hCG), and CT scan of the abdomen). Specialist on call surgeons were in charge to decide for operations,

### Inclusion criteria

Alvarado scoring applied for all, when it was six and more scores, decision was in favor of appendectomy, all appendectomies were by open method, no laparoscopic appendectomy was done as it was not available at that period in the emergency department.

### Exclusion criteria

While those patients who were, improved and have Alvarado scoring 5 or less, kept under observation for up 24 hours and discharged well without operations were excluded as well as those patients (on decision of the team of surgeon and gynecologist), who were sent to gynecology and obstetrics hospital for gynecological or obstetric conditions were excluded.

Perioperative antibiotics (Ceftriaxone 500 mg in a drip given intravenously), and preemptive analgesia (500 mg acetaminophen suppository) were prescribed for each patient.

Operative data regarding state of appendices, histopathological results of appendices, and post-operative morbidity and mortality up to one month were collected and organized according to international code of diseases.

All the collected data were collected, organized, then analyzed by Statistical Package for the Social Sciences (SPSS); version 21.

## Conclusion

Negative appendectomies has high rates of morbidity and mortality, knowing real rates may help in considering various policies i.e. re-examination, rescanning, waiting few hours for observation of equivocal cases, considering antibiotics as initial treatment of selected cases of suspected appendicitis, may be helpful to elude avoidable complications and potential mortality.

### Strength and Limitations of the study

#### Strength

- Clear massage, not to be hasty in surgery for suspected acute appendicitis, give time for longer observation, and to be open for new notion of conservative treatments of some patients with suspicion of acute appendicitis.
- This will help in providing the high level of evidence to acquire a better evidence-based decision making on appendicitis.

### Limitations of the study

- The decision for appendectomy was not from one surgeon, albeit from all on call surgeon in Emergency Department, this may be a bias factor, as all are not of same level of experience, their decision logically will be different.
- Near half of the patients presented to the Emergency department out the hours where imaging and specially CT scan and MRI were not available (from 20:00 to 08:00), to help in clarification of diagnosis in equivocal and complicated cases of suspected acute appendicitis. Only 10 patients were sent for CT scans which change the clinical decision of the surgeon for appendectomy: CT scan of two patients were negative for appendicitis, treated conservatively and had smooth course, with no morbidity or mortality. While CT scan of eight patients were positive for acute appendicitis, then underwent appendectomy with smooth postoperative course, with no morbidity and modalities. No patients had high clinical suspicion of appendicitis but negative CT scans.
- The data is not available on follow up of the patients who were discharged after 24 hours of observation patients, to add strength to study.

### Compliance with ethical standards

The work is compliance with Ethical Standards.

### Ethical approval

It was approved by Ethics Committee of college of Medicine, University of Sulaimani (No. 2, on 8^th^, November 2013).

### Informed consent

All methods were performed in accordance with the relevant guidelines and regulations, and informed consent was explained and signed by each patient and senior house officer on duty in face to face interview.

## Data Availability

Authors do not wish to publicly share the data, Please contact author for data requests.
